# Ultraconformable Temporary Tattoo Electrodes for Electrophysiology

**DOI:** 10.1002/advs.201700771

**Published:** 2018-01-03

**Authors:** Laura M. Ferrari, Sudha Sudha, Sergio Tarantino, Roberto Esposti, Francesco Bolzoni, Paolo Cavallari, Christian Cipriani, Virgilio Mattoli, Francesco Greco

**Affiliations:** ^1^ Center for Micro‐BioRobotics @SSSA Istituto Italiano di Tecnologia Viale Rinaldo Piaggio 34 56025 Pontedera Italy; ^2^ The BioRobotics Institute Scuola Superiore Sant'Anna Viale Rinaldo Piaggio 34 56025 Pontedera Italy; ^3^ Human Physiology Section of the De.P.T. Università degli Studi di Milano Via Mangiagalli 32 20133 Milano Italy; ^4^ Department of Life Science and Medical Bioscience Graduate School of Advanced Science and Engineering Waseda University 2‐2 Wakamatsu‐cho, Shinjuku‐ku 169‐8480 Tokyo Japan; ^5^ Institute of Solid State Physics Graz University of Technology Petersgasse 16 8010 Graz Austria

**Keywords:** conformable materials, electrophysiology, epidermal devices, inkjet printing, temporary tattoos

## Abstract

Electrically interfacing the skin for monitoring personal health condition is the basis of skin‐contact electrophysiology. In the clinical practice the use of stiff and bulky pregelled or dry electrodes, in contrast to the soft body tissues, imposes severe restrictions to user comfort and mobility while limiting clinical applications. Here, in this work dry, unperceivable temporary tattoo electrodes are presented. Customized single or multielectrode arrays are readily fabricated by inkjet printing of conducting polymer onto commercial decal transfer paper, which allows for easy transfer on the user's skin. Conformal adhesion to the skin is provided thanks to their ultralow thickness (<1 µm). Tattoo electrode–skin contact impedance is characterized on short‐ (1 h) and long‐term (48 h) and compared with standard pregelled and dry electrodes. The viability in electrophysiology is validated by surface electromyography and electrocardiography recordings on various locations on limbs and face. A novel concept of tattoo as perforable skin‐contact electrode, through which hairs can grow, is demonstrated, thus permitting to envision very long‐term recordings on areas with high hair density. The proposed materials and patterning strategy make this technology amenable for large‐scale production of low‐cost sensing devices.

## 1 Introduction

The recent surge of “epidermal electronics”^1^ field has been enabled by advancements in technology of electronic materials: devices go thinner and more flexible (or even stretchable) than ever.^2^, ^3^, ^4^, ^5^ These innovations have seen the introduction of organic electronic materials in the portfolio of materials scientists and electronic engineers, besides the well‐known inorganic semiconductors, metals, and oxides.^6^, ^7^ Progresses in printed electronics provided suitable techniques for fabrication of devices above novel plastic or paper substrates with large‐area/high‐throughput capability.^8^, ^9^ On the other hand, interfacing devices with skin can open new pathways for applications, like the development of unprecedented personal health monitoring systems, sensors, drug delivery systems, among the others.^10^, ^11^, ^12^ Indeed, electrically interfacing with the outer layer of our body—the skin—can provide important information about the health condition, through the monitoring of chemical and physical variables. For instance muscles, brain, or heart activity, as well as other vital parameters,^5^, ^13^, ^14^, ^15^ could be tracked and appropriately processed. Skin‐contact electrophysiology, with its well‐established and multifacet techniques—such as electromyography (EMG), electrooculography, electrocardiography (ECG), electroencephalography (EEG)—consists in the recording of the electrical activity related to functioning of cells, tissues, organs, with the principal purposes of diagnosis and monitoring. By means of electrodes placed on the skin, voltage or current recording permits to analyze the functioning of different tissues and organs, at different resolution levels, depending on the technique and number/density of electrodes used. Most skin‐contact electrodes in use for clinical practice or research (Ag/AgCl) operate with an electrolytic gel for establishing a stable electrode–skin wet interface.^16^, ^17^ Although their intensive use, reported drawbacks of wet (gelled) electrodes are: limited signal stability over time (as the gel dries out),^18^ short circuit in high density recording (due to the gel leakage across neighboring electrodes), discomfort, heft/rigidity against skin compliance, and the requirement for skin cleaning/preparation. In the quest for unobtrusive, lightweight yet reliable electrodes interfacing with skin we recently proposed conducting polymer‐based temporary tattoo nanosheets.^19^ Temporary tattoo paper was used as an unconventional substrate for electrodes fabrication. This substrate included a thin (around 500 nm thickness) ethylcellulose (EC) layer on top of which a layer for poly(3,4‐ethylenedioxythiophene):polystyrene sulfonate (PEDOT:PSS) was spin coated. The tattoo electrode was then transferred onto the target surface (skin) by dissolution of a water‐soluble sacrificial layer. The same substrate has been recently proposed for fabricating skin‐worn sensors for metabolites.^5^, ^20^, ^21^ However, in order to meet the requirements toward practical applications, like vital parameters monitoring in healthcare and sport, such tattoo electrode technology needs to be advanced. On the one hand, patterning of electrodes is strongly required, as multielectrode array tattoo is needed in any of the mentioned applications. Moreover, integration of stable interconnections and external connectors is mandatory, while maintaining an easy and reliable transfer on the skin. The main challenge is to optimize functionality while retaining ultralow thickness and flexibility, thus enabling a seamless interface.^22^, ^23^, ^24^ Lately, Hanein and co‐workers^25^ elaborated on a similar strategy as Zucca et al.^19^ and developed patterned electrodes on temporary tattoo paper by using screen printing of carbon ink followed by plasma polymerization of PEDOT:PSS. Recording of EMG signals and operation up to 3 h were demonstrated. However such tattoo electrodes were relatively thick (substrate 1.2 µm, overall thickness of electrodes around 100 µm), thus preventing a conformal contact with the complex texture of skin, made of 10–100 µm thick valleys and ridges. Recently, a “wet transfer, dry patterning strategy” has been proposed for ultrathin graphene electronic tattoo sensors,^26^ in which graphene is supported on a thin (around 440 nm) polymethylmethacrylate or thicker (around 13 µm) polyimide layer. These are first transferred on tattoo paper (few µm thick) or medical adhesive (few mm thick), cutted out in the form of ribbons, then transferred on skin by wetting the tattoo paper, and used for electrophysiological recordings. Actually seamless interfacing is sought in skin‐contact electrophysiology applications, in order to improve the quality of the recording. An ultrathin, therefore ultraconformable, sensor interface can maximize the contact area (hence signal amplitude) and may avoid movement artifacts arising from the relative displacement between skin and electrodes. Moreover, comfort for the user and long‐term stability are important requirements especially in long‐term recordings or whenever complex or delicate anatomical districts have to be investigated (e.g. the face). Intrinsic obtrusiveness and weight of the electrodes severely curtail such recordings, and can hamper or modify the natural skin stretching/displacement caused by the activation of underlying muscles. As a matter of fact, an unperceivable electrode technology can open new possibilities for skin‐contact sensing, both for clinical diagnosis/monitoring and for research. In order to address the above‐mentioned requirements and starting from our previous results, we elaborated a strategy for the fabrication and multilayer assembling of temporary single or multielectrode tattoo. Inkjet printing was adopted for fabrication, as this noncontact manufacturing method can allow for low‐cost, large‐area, reliable, high resolution device production, and possibly future integration with other printed electronic components.^9^ In this paper we present the materials and processes used to manufacture various kinds of ultrathin and ultraconformable single electrode tattoos and temporary tattoo multielectrodes arrays (TTMEAs), suitable for different electrophysiological measurements. Their thickness, electrical properties, stability, and interface with skin are assessed and discussed. In particular, the electrode–skin contact impedance of the tattoo electrodes is compared with state‐of‐art electrodes. Electrode–skin contact impedance was recorded up to 2 days, and its substantial stability was demonstrated. This suggests the use in long‐term continuous applications, e.g. monitoring of vital parameters. Several proofs of concept for application in face or limb EMG, as well as ECG are presented and discussed. Finally, we demonstrate hair growing through tattoo electrodes over 24 h, with no visible effect on the quality of the recording. This result allows to envision unprecedented applications, e.g. long‐term monitoring of EEG activity on shaved scalp, which is currently not possible with wet (gelled) electrodes due to the drying of the electrolyte and eventual displacement of the electrodes caused by hair growing.

## 2 Results and Discussion

### 2.1 Fabrication and Characterization of the Temporary Tattoo Electrodes

A careful selection of materials, processing, and layer assembling techniques was carried out to meet the various requirements for obtaining temporary tattoo skin‐contact electrodes with integrated internal and external connections. First, in order to retain the advantages of using temporary tattoo paper as a substrate for electrodes, it was necessary to select compatible materials. Indeed the substrate, EC layer on decal paper, is low‐cost, skin safe, easy to use, and available in large area. Second, to get full advantage of said benefits, manufacturing methods (inkjet printing, cutting, and lamination) were selected, since they allowed for low‐cost and large‐area device production. Moreover such methods enable future integration with other printed electronics components. As this investigation was aimed to fabricate and test skin‐contact electrodes, it was mandatory to consider thickness as a feature of paramount importance. Indeed in epidermal electronic devices optimal mechanical properties are given by a proper combination of effective modulus and thickness, in order to interface with skin and minimize delamination/ruptures.^27^ These properties reflect in devices inherent bending stiffness, determining their ability to flex to small curvature radius, following the skin relief profile in conformal adhesion. In the present case the main constituents of tattoos are polymers, having Young's modulus in the range of ≈1 GPa (EC ∼ 0.8–1.3 GPa;^28^ PEDOT:PSS ∼ 0.8–1.1 GPa^29^), and the overall thickness is submicrometric. These two peculiar characteristics were selected with the aim of getting conformal adhesion to the skin.

#### 2.1.1 Single Electrode Temporary Tattoo

**Figure 1:**
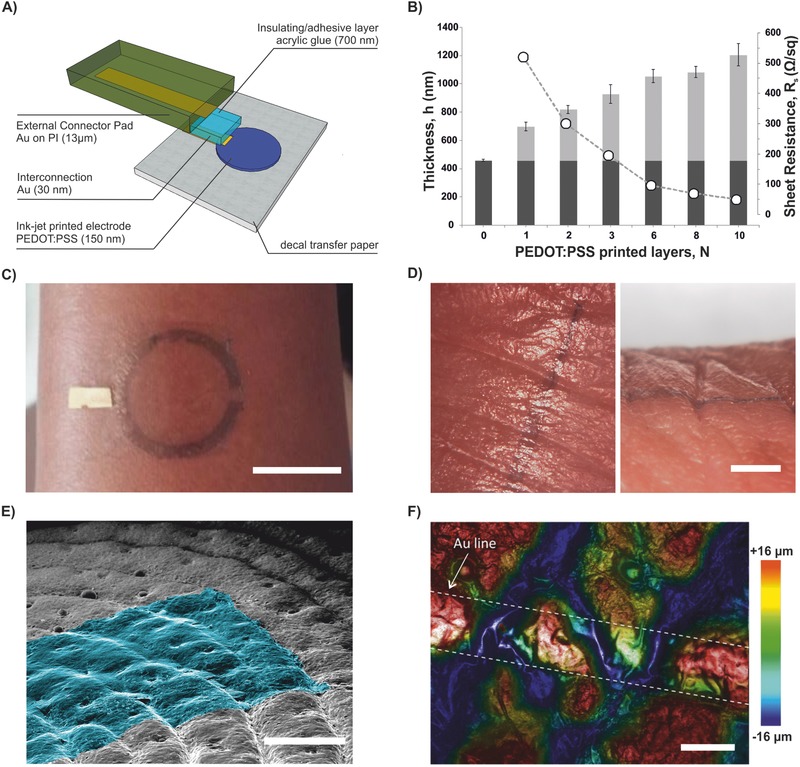
Multilayer assembly of temporary tattoo electrode and conformability of tattoo electrodes onto human skin: A) Layers schematic 3D exploded view. B) Overall thickness of electrodes (*h*, EC layer in dark gray, PEDOT:PSS layer in light gray) and sheet resistance (*R*_s_, circles) as a function of number of printed layers (N). C) Assembled circular electrode transferred on the arm (scale bar = 2 cm). D) Optical microscope pictures of tattoo with PEDOT:PSS printed line (*w*: 0.50 mm) transferred on the forefinger and conformally following the profile of deep folds in skin: top view (left) and side view (right) (scale bar = 2 mm). Tattoo transferred onto silicone replica of human skin: E) colorized SEM micrograph (45° tilted view, scale bar = 400 µm); F) optical profilometer, superposition of bright field, and topography images showing topography of an Au line (i.e. interconnection, *w*: 200 µm) on tattoo (scale bar = 200 µm).

Single temporary tattoo electrodes were based on a multilayer design (**Figure** **1**A). Skin‐contact electrodes were realized by inkjet printing on tattoo paper of a waterborne PEDOT:PSS dispersion. Interconnecting lines (Au) joined the electrodes with the external connectors, the latter being made by flat polyimide (PI) strips laminated on tattoo paper with acrylic glue sheet layer where necessary (see also Figure S1, Supporting Information). The overall thickness of tattoo electrodes (EC+PEDOT:PSS) and, consequently, their sheet resistance were varied as a function of printed layers of PEDOT:PSS (Figure 1B). While substrate EC layer had a constant thickness of 450 nm, overall electrode thickness ranged from h ∼ 600 to ∼1200 nm, with a consequent drop of sheet resistance from *R*_s_ = 520 to 50 Ω sq^−1^. Assembled electrodes could be transferred onto skin (Figure 1C) by gently pressing the tattoo against the skin and wetting the supporting paper, which caused the dissolution of sacrificial starch layer (Movie S1, Supporting Information). Temporary tattoo electrodes showed stable conformal adhesion onto skin over the whole range of thickness tested and on several locations on the body: arm, face, legs, chest, and fingers (Figure 1C,D). The capability of the tattoos to conformally adhere to the creased surface of skin was even better evidenced at the microscopic level (Figure 1E,F): tattoo electrodes were able to enter deeply inside creases, thus maximizing the sensing area and interfacial adhesion/stability. Because of the ultralow thickness and negligible weight of the electrodes they did not induce any apparent constraints in the user motion so that skin could deform freely and reversibly (Movie S1, Supporting Information). Volunteer users which worn tattoo on skin for different tests (11 persons) were asked to describe whether they perceived or not the presence of tattoo and if their movements were somehow affected or hampered by tattoo. In all cases users described not to perceive the electrode but had perception of the connector part (wire or flat connector, placed at the edge of tattoo); indeed this part is thicker/stiffer than the sensing part. However users did not describe any constraint of their natural movements. After their use, the temporary tattoo electrodes could be removed by washing with soap and water and gently rubbing, as in the case of temporary tattoo for children. Skin irritation was not observed even in the case of tattoos worn on skin for long‐term recordings (2 days—see below). In just one case, a subject showed moderate skin irritation to the EC layer (usually adopted in commercially available temporary tattoo and approved for skin‐contact use), and thus probably indicating a subjective and uncommon sensitivity of skin to this material.

Single electrode tattoos were subjected to stretching tests in order to assess the maximum range of stretching they can sustain before failure. The results (Figures S2 and S3, Supporting Information) permitted to appreciate a good recovery up to 5% strain, as expected based on previous findings on PEDOT:PSS.^30^, ^31^ A first failure is observed at around 8%, probably ascribable to failure of wire interconnection rather than to tattoo integrity. The latter seems to be maintained even after repeated cycles at 10% of strain as visible cracks or delamination is not observed in the electrode area (Figure S3b, Supporting Information).

**Figure 2:**
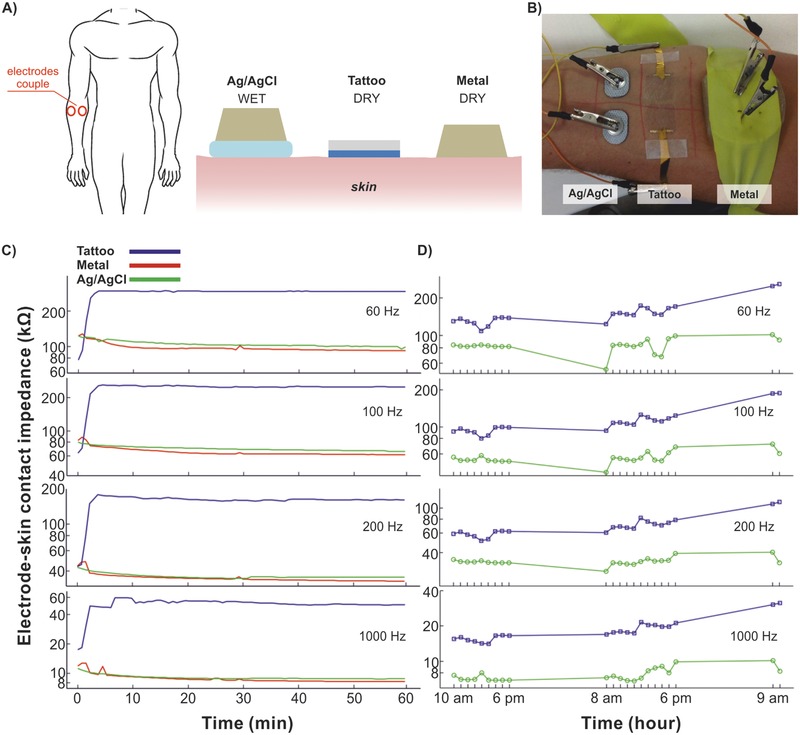
Characterization of tattoo electrodes electrical interfacing with skin: impedance across couple electrodes. A) Schematic of the three electrode types: Ag/AgCl, tattoo, and metal. B) Picture of the three pairs of electrodes on the forearm. C) Short‐term test: evolution of the median impedances (*n* = 7) at representative frequencies over 1 h. D) Long‐term test: evolution of the Ag/AgCl and tattoo impedance (*n* = 1) at representative frequencies over 48 h.

*Short‐ and long‐term impedance assessment*: The 1‐h short‐term experiments demonstrated the temporal trends of three types of electrodes: tattoos, Ag/AgCl, and dry (**Figure** **2**A,B). The impedance of the tattoo electrode quickly increased and stabilized in the first few minutes after application. This behavior is likely to reflect the drying of the water used to apply the tattoo on skin. Instead, in the following 55 min the impedance was practically constant at all spectrum values (Figure 2C—only components at 60, 100, 200, and 1 kHz are displayed). The impedance of the dry and of the Ag/AgCl electrodes proved comparable (Figure 2C), thus suggesting the presence of humidity under the dry electrode due to the sweating of the skin, as reported earlier by Geddes et al.^32^ The average modulus of the impedance at 60 Hz after 60 min was 294 ± 1 kΩ (median ± Inter Quartile Range) for the tattoo, 94 ± 3 kΩ for the dry, and 102 ± 2 kΩ for the Ag/AgCl electrode. The latter values matched those assessed by Searle and Kirkup when testing dry and Ag/AgCl electrodes.^17^ To our knowledge this is the first time that the short‐term time evolution of the impedance was recorded in a dry conformal electrode, hence no comparison can be made with other electrodes. In the EMG frequency domain (10–450 Hz, where the EMG shows a high power density) the tattoo electrodes exhibited an impedance below 300 kΩ being ∼300 kΩ at 60 and 100 Hz, similar to other conformal electrodes^13^, ^18^, ^25^ The comparison among the electrodes revealed that tattoos exhibited larger impedance, at all frequencies greater than 10 Hz (Figure S4A, Supporting Information), thus suggesting a shifted pole compared to conventional (titanium and Ag/AgCl) electrodes. Notably, the PEDOT:PSS is an antistatic material and this likely reduces the capacitance associated to the electrode–skin interface. The comparison between the dry and Ag/AgCl impedance did not exhibit differences across all the relevant spectrum (Figure S4A, Supporting Information), in agreement with the available literature.^32^ The results from the long‐term experiment were in substantial agreement with the short‐term trial, both analyzing the temporal evolution at relevant frequencies (Figure 2D) and the spectrum over time (24 and 48 h after the application; Figure S4B, Supporting Information). Remarkably, the test proofed the possibility of recording for 48 h, with the tattoo electrodes and the pregelled ones. The temporal stability was found relatively constant for the duration of the experimental trial, along the entire spectrum. In particular this was true for the impedance at 1 kHz, in agreement with the results by Leleux and co‐workers, who used ionic liquid gel‐assisted electrodes.^18^ A moderate increase of the impedance with time and a scattered trend was observed (Figure 2D). The relatively scattered trend, recorded from both kinds of electrodes, was likely due to a multitude of reasons. For example, physiological changes (e.g. the hydration of skin) of the tested subject, as well as environmental conditions (e.g. humidity and temperature). Unfortunately it was impossible to control these variables in the 48‐h test, as reported by other investigators.^33^ Notably, in contrast with the results reported by Leleux et al.,^18^ we did not observe the drying of the Ag/AgCl electrodes after the first 12 h. However, this difference may be ascribed to the different electrodes used and perhaps to different environmental conditions difficult to track.

*Surface electromyography (sEMG) and ECG recordings with single electrodes*: Tattoo electrodes capability to act as electrophysiological transducers was tested in surface electromyography (sEMG) and ECG. Regarding sEMG, pairs of tattoo and of Ag/AgCl electrodes were placed over the *biceps brachii* of a volunteer for comparison; three different contraction levels were produced (see ‘Materials and Methods' section and Figure S5 in the Supporting Information). The signals gathered from the two kinds of electrodes were comparable at all contraction levels.

**Figure 3:**
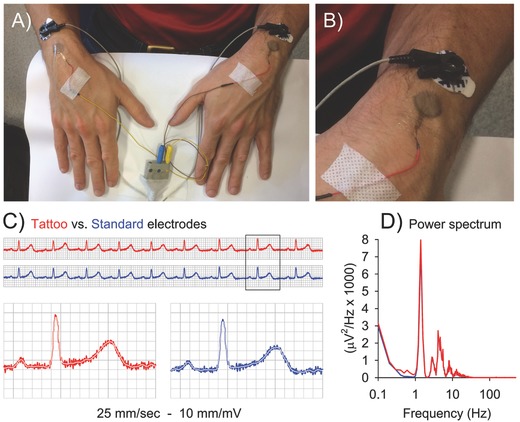
Comparison of lead I ECG simultaneous recording, performed with tattoo and standard electrodes. A and B) Experimental set‐up, tattoo, and standard electrodes having the same active area (diameter 16 mm). C) Sample of continuous recording (top) and sample of a single beat (bottom), obtained by tattoo (red) and standard (blue) electrodes. White line depicts the 40 Hz low‐pass filtered trace. D) Power spectrum of the two signals along 50 s recordings.

In the case of ECG, tattoo and standard gelled electrodes were placed on both wrists of a volunteer (**Figure** **3**A,B). The ECG signal was simultaneously recorded for 50 s consecutively (a 6 s sample is shown in Figure 3C) and compared, also in terms of power spectrum (Figure 3D).

Being impossible to compare hearth activity to “rest”, the “noise” RMS amplitude was estimated as the square root of the integral of the power spectrum above 40 Hz, where the ECG signal has no components. The noise RMS amplitude resulted to be 16.8 µV with tattoo and 14.0 µV with standard electrodes. The total RMS amplitude was instead 108.4 versus 105.2 µV, giving a signal‐to‐noise ratio of 6.45 and 7.52, respectively. Results suggest that the tattoo electrodes can be safely used in place of standard electrodes.

Meaningful ECG recordings could be also obtained by directly connecting tattoo electrodes to a portable low‐cost ECG monitor, demonstrating their use in market‐available personal monitoring devices (Figure S6, Supporting Information).

#### 2.1.2 Temporary Tattoo Multielectrodes Arrays (TTMEAs)

**Figure 4:**
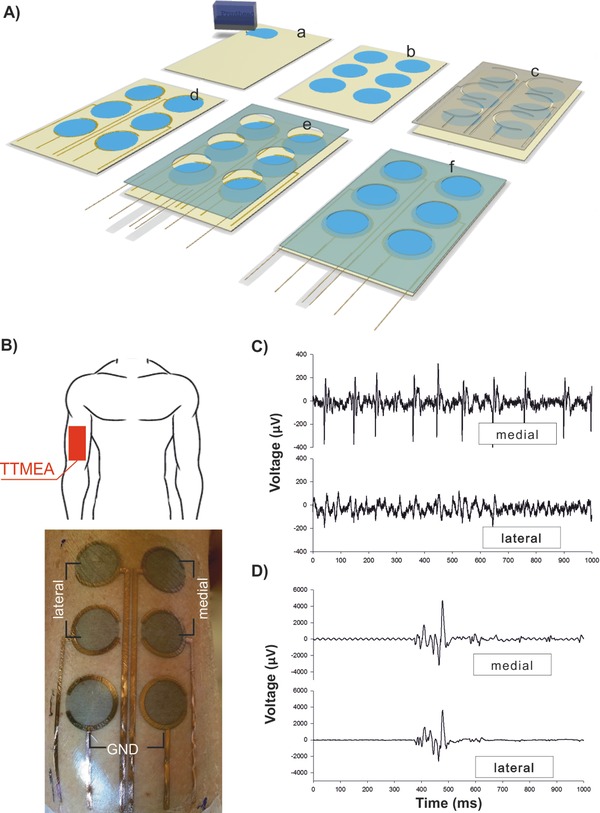
Temporary tattoo multielectrodes arrays (TTMEAs) for sEMG recording. A) Schematic view of the TTMEAs fabrication flow divided in six steps. (a and b) Inkjet printing on the tattoo paper of the PEDOT:PSS sensing area (circle, *d*: 16 mm). (c and d) Mask and Au sputtering deposition for internal interconnections. (e and f) Assembling of the whole tattoo with shaped glue layer and wires for external contact. B) Schematic of location of TTMEA (top) and picture of TTMEA transferred on the right biceps of a voluntary subject (bottom). The array design has been chosen in order to detect signals from two different channels: medial and lateral; the bottom pair of electrodes is the ground references (highlighted in picture). C) EMG recordings during a very low isometric contraction, aimed at recruiting few motor units; the apparent repetitive firing in the upper trace, but not in the lower one, is produced by one single motor unit, indicating the high selectivity of the recording. D) A single burst of voluntary phasic contraction is almost equally recorded by both channels. In (C and D), the signal was filtered in a 10 Hz–3 kHz frequency window and notch line filter was off.

**Figure 5:**
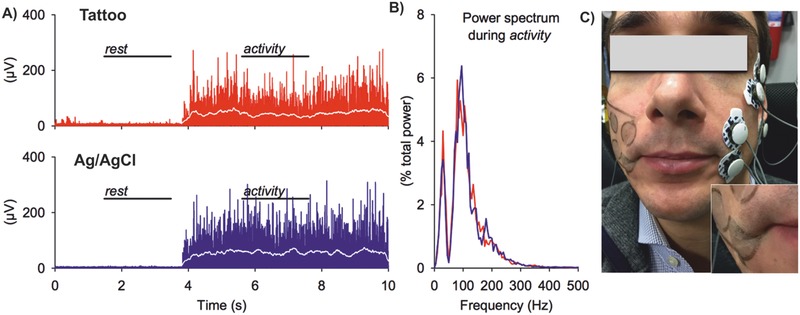
sEMG recordings on the face. A) Rectified EMG (raw signal in red/blue, integrated for 200 ms in white) from tattoo electrodes (red) on the right corner of the mouth and from Ag/AgCl pregelled electrodes (blue), on the left side. B) Power spectrum of the right and left EMG in the activity period, normalized to the respective total power. The hole at 50 Hz is due to the notch filter. C) Picture of subject wearing tattoo and Ag/AgCl electrodes on face. Inset shows detail of tattoo electrodes placed at right corner of mouth and its capability to follow the complex topography of face during muscle contraction.

Multiple electrodes arrays (MEAs) are often used in electrophysiology recordings as they can allow the placement on skin of multiple electrodes at once, with known and fixed relative position each other, and with the possibility to choose among various recording channels. MEAs were fabricated onto temporary transfer tattoo as the evolution of the single electrode design (**Figure** **4**A), as described in detail in Supporting Information. It is important to note that, as their fabrication was based on inkjet printing, the design of such TTMEAs (electrode shape, number, surface, spacing, arrangement) can be modified in order to meet the different requirements for the particular application and target location on the body. Figure 4A shows an example of 6‐electrodes MEA. A different design is shown in Movie S1 (Supplementary Information). sEMG recordings with TTMEAs were tested both on the biceps and on the cheek of voluntary subjects. The results obtained in the case of biceps (Figure 4) confirmed the ability of the electrodes array to record specific EMG signal, of good quality both in isometric and during phasic contraction. In low intensity isometric contraction, isolated firing was specifically detected by the electrode pair overlooking the recruited motor unit. For example, periodic spikes were apparently recorded by the medial electrode pair but not by the lateral one (Figure 4C). Also phasic activity recordings exhibited good quality, allowing the acquisition of brief explosive events (bursts, Figure 4D), equally well visible in both traces. Due to their low thickness and ultraconformability, the tattoo electrodes are suitable for electrophysiological recordings on anatomical districts (e.g. skin folds on face) which are difficult to address with currently available technologies. With the aim to verify the suitability of the proposed approach, TTMEAs were simultaneously tested against standard pregelled electrodes on the orbicularis oris muscle group. For tattoo electrodes (on the right cheek), RMS amplitude during rest (marked by leftmost horizontal line in **Figure** **5**A) was 3.0 µV, while during tonic symmetric bilateral activity (rightmost line) reached 50.6 µV. Values obtained with standard electrodes (on the left cheek) were: RMS amplitude 1.9 µV in rest and 72.5 µV during activity (Figure 5B). Figure 5C shows the power spectrum during activity. Note that despite the lower signal‐to‐noise ratio (activity RMS/rest RMS) of the Tattoo versus Standard electrodes signals (16.9 vs 38.1), the two spectra were at all comparable. The median frequency was indeed 96.6 Hz for the Tattoo versus 96.1 Hz for the Standard electrodes, while the mean frequency was 112.4 Hz versus 112.6 Hz, respectively. The hole at 50 Hz is due to the notch filter on the EMG amplifier.

### 2.2 Hair Growing Through Temporary Tattoo Electrodes

**Figure 6:**
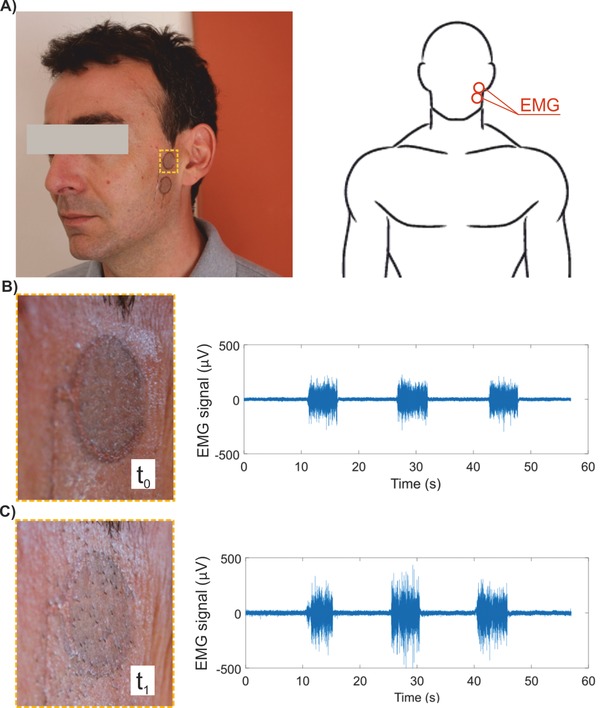
Facial hair growing through tattoo electrodes. A) Two circular tattoo electrodes were placed over the mandibular muscle of a subject at time *t*_0_ = 3 h after shaving. The user worn the tattoo electrodes for 24 h up to *t*_1_ = 27 h to assess functionality after hair have grown through the tattoo electrodes. B and C) Detail of tattoo at *t* = *t*_0_ , *t*_1_, and corresponding sEMG recordings.

As a relevant part of human body is covered with hair, and these represent a physical obstacle for electrically interfacing skin, many electrophysiological recordings require shaving. Moreover hair growth rate is relatively fast (typically 0.3–0.5 mm day^−1^ in scalp; the actual rate depending on hair location, age, sex, race, and health state^34^), therefore a long‐term recording with standard electrodes is often impossible or limited. Indeed, even if the target surface is shaved before electrodes placement, growing hairs, pushing against electrodes, cause poor electrical interfacing and even electrode displacement over time. These issues can be particularly relevant when recordings are needed on areas with high follicle density, such as the scalp. As an example, EEG long‐term recordings, with duration of several days, are actually prevented for this reason. The peculiar ultralow thickness and conformability of temporary tattoo electrodes permitted us to envision a capability that is impossible in all other available technologies. We investigated whether a growing hair can punch through the tattoo electrode without causing its delamination or impairing its functionality. To this aim, we placed a couple of tattoo electrodes on the shaved face of a subject (**Figure** **6**A). A first EMG recording of mandibular muscle contraction was taken at *t*_0_, 3 h after shaving (Figure 6B). EMG recording was repeated after several time intervals, with good stability and no evident signal degradation despite the fact that hair grew through tattoo electrode. The experiment lasted for 24 h, up to *t*_1_ (Figure 6C). The test was made with facial hair for obvious convenience, but a similar hair punching is expected on the scalp. The average hair density on scalp is reported as *N*_h_ ∼ 160 hairs cm^−2^;^2^, ^34^ given an average hair diameter *d* = 60 µm, and considering the section of hair as a surface which is not available for electrical recording, this results in a “hair occupied surface” of around *S*_h_ = π(*d*/2)^2^ *N*_h_ = 4.5 × 10^−3^ cm^2^, per cm^2^, thus about 0.5% of scalp surface, which is negligible with respect to the electrode area.

## 3 Conclusion

The results presented so far provide a comprehensive characterization of tattoo electrodes as regards their structure, their conformal adhesion and their dry electrical interfacing with skin. The use of dry contact eliminates drawbacks arising from a gel layer and its stability over time. With an overall thickness lower than 1 µm, we presented the thinnest skin‐contact electrodes to the best of our knowledge. This guaranteed excellent unperceivability. Impedance records highlighted the adoption of tattoo electrodes over long term and with performance comparable with standard electrodes. EMG and ECG recordings permitted to appreciate good recording capabilities in combination with dedicated laboratory measurement setup and also with commercially available portable devices. The adopted manufacturing method, based on inkjet printing and lamination, enables to fabricate multielectrodes arrays with freedom in design. The latter is desirable in view of specific applications in diagnostics, human machine interfacing and personal health monitoring in sport, among others. Based on EMG records, TTMEAs can be thought as ideal electrodes for facial recognition; indeed, soft and lightweight tattoo electrodes don't modify natural movements of users as in the case of bulky electrodes. Future directions of this research should also consider recording of long‐term EEG on scalp, which is impossible to perform with standard electrodes due to the displacement of electrodes during hair growth. On the contrary, tattoo electrodes can be perforated by hairs growing through it without a visible loss of recording capability, and remain stably adhered to skin. While two different strategies for wired connection with external devices are presented, further efforts should be dedicated to establish wireless communication, e.g. through printed RF antenna and analogic front‐end on the same tattoo substrate.

## 4 Experimental Section

*Materials*: PEDOT:PSS aqueous dispersion (Clevios PJet 700 by Heraeus) had been used to fabricate the sensing part of the electrodes. Commercially available temporary transfer tattoo paper kit (Tattoo 2.1, by The Magic Touch Ltd., UK), composed of two sheets, decal transfer paper and glue sheet, had been employed as unconventional substrate and passivation layer, respectively (Figure S1, Supporting Information). The decal paper, used as substrate for electrodes fabrication, was made up of three layers: a paper carrier, a starch–dextrin water soluble layer, and an EC (thickness ≈ 450 nm) layer. The glue part was a three‐layered sheet composed of a silicone paper carrier sheet, acrylic glue (thickness ≈ 700 nm), and a plastic liner. The glue part was used for providing tattoo adhesion while preventing direct contact of interconnection lines with skin. Polyimide film (Kapton by Goodfellow, thickness 13 µm) had been employed as support layer for the external electrical connection, in case of planar connectors. Standard acrylic transparent sheet (thickness 0.3 mm) was used to build up laser‐cut masks.

*Fabrication*: Fabrication process included inkjet printing of electrodes and assembling of laser cut layers through a lamination process. Inkjet printing was carried out with a Dimatix DMP‐2800 system (Fujifilm Corp., Japan) endowed with a 10 pL cartridge (DMC‐11610). PEDOT:PSS ink was used for inkjet printing after filtration (Minisart, average pore size 0.20 µm, Sartorius). The lamination process allowed the assembling of multiple layers, each layer having an internal set geometry fabricated through laser cutting. A CO_2_ laser cutter (VLS 3.50, 50 W; Universal Laser Systems) was used both for the production of masks and the shaping of each layer, while a high vacuum sputtering system (Q150T S Turbo‐Pumped Sputter Coater, Quorum Technologies Ltd.; 70 W, 2 × 10^−2^ argon pressure and 1 min deposition time for 30 nm thickness) was adopted for the deposition of Au interconnecting lines. In particular two different configurations of tattoo electrodes were realized: single and multielectrode array. Detailed procedures for fabrication and assembling of the different layers and external connectors are given in the Supporting Information.

*Surface Characterization*: Surface analysis was performed on tattoo samples, recollected onto different specific supports, after the release in water as free standing membrane (by the dissolution of starch layer). Thickness measurements were carried out with a P6 stylus profilometer, KLA Tencor, onto samples recollected on clean Si wafer. SEM micrographs were obtained with a FEI HELIOS 600 Nano Lab Dual Beam, operating at 5 KV, on samples recollected onto silicone skin replica. Topographical images were obtained with an optical profilometer (LEICA DCM 3D optical profiler) operating in confocal mode on a tattoo sample recollected onto silicone skin replica.

*Experiments Involving Human Subjects, Participants, and Experimental Procedure*: Nine able‐bodied subjects (5 males and 4 females aged 29.5 ± 3.5 years old) free of any motor disorders participated to this study. Informed consent in accordance with the Declaration of Helsinki was obtained before conducting the experiments from each subject. Seven participants volunteered in the experimental measurement of the electrode–skin contact impedance of three types of electrodes measured for 60 min. One of these participants took part in a 48‐h long test, during which the impedance of two types of electrodes was periodically measured. Another participant performed a comparison test in which the quality of the signals recorded by the tattoo electrodes was correlated with those picked up by common Ag/AgCl pregelled electrodes. One of the volunteers was selected for ECG recordings, with pregelled and tattoo electrodes. Another volunteer was selected for the hair growth experiment. Finally, two other participants took part in qualitative tests for TTMEAs evaluation on different body district, arm, and face.

*Short‐Term Impedance Assessment (60 min)*: To examine the influence of electrode construction on contact impedance, the tattoo electrodes were compared over time with two other kinds of electrodes when applied to the forearm (anterior surface) (Figure 2A,B). As the impedance of the bulk tissue between the electrode pairs could be considered equivalent for all couple of sensors, a fair comparison was possible.^35^ The control electrodes were: disposable pregelled Ag/AgCl (by Spes Medica, model DENIS01520) and titanium dry electrodes, all having the same active surface (300 mm^2^). The interelectrode distance as well as the interpair distance were set to 20 mm. The participant sat comfortably on a chair, with his/her forearm lying supinated on a bench. Using the technique described by Searle and Kirkup^35^ the impedance between each pair of electrodes was recorded over 60 min, using a custom circuit (based on the OP177) and a PC equipped with a data acquisition board (NI PCIe‐6259, National Instrument, Austin, TX, USA). For the impedance monitoring a multiplexing system was designed to apply a harmonic current stimulation to each electrode pair in turn. In this way the impedance for all electrodes (at 15 frequencies over a range of 2–1000 Hz) was recorded every 4.5 s, immediately after the application of electrodes. For each recording the modulus of each spectral component of the impedance was computed as the average across the seven subjects. Special attention was paid to the bandwidth of interest of the EMG signal, i.e. from 10 to 450 Hz.^16^

*Long‐Term Impedance Assessment (48 h)*: The evolution of the tattoo electrode–skin impedance with time was further assessed over a 48 h time, using the same recording equipment of the short‐term test. Only the Ag/AgCl pregelled electrodes could be tested for comparison, since the titanium dry electrodes were assembled on an uncomfortable support that could not be used overnight. The electrodes were placed on the same spots used for the short‐term test (Figure 2 A,B) and maintained for 48 h. The participant was asked not to (deliberately) touch/wet the electrodes; other than this, she had no other restrictions. A soft bandage was used to protect the electrodes, between recordings and overnight. For each measurement session, the modulus of each spectral component of the impedance was computed as the mean across the 14 recordings.

*ECG Recording*: In order to have a parallel recording, a pair of tattoo electrodes (circular shape, sensitive area diameter 16 mm) and a pair of standard gelled electrodes (Kendall H124SG, circular shape, sensitive area diameter 16 mm) were closely placed on the wrists of a healthy subject. Signals were amplified (gain 1 k), filtered (0.1–300 Hz, with a notch at 50 Hz), and sampled (1 kHz, 12 bit) for 50 s. Power spectrum was calculated on raw sampled data by the Welch's method (10 s epochs, 50% overlap, Hanning window). The “noise” RMS amplitude was estimated by integrating the power spectrum above 40 Hz, where the normal ECG signal had no components, and extracting the square root. The ratio between the total RMS and the “noise” RMS amplitude was used as a signal‐to‐noise ratio estimate.

In a second experiment, electrodes were directly connected to an ECG portable device (Figure S6, Supporting Information). The ECG monitor was a market available, easy to use device, which could be operated by nonspecialized users (Prince 180 B, by Heal Force).

*sEMG Recordings with TTMEAs*: sEMG recordings with TTMEAs were tested either on the biceps or on the cheek of voluntary subjects. In both cases a 6‐electrode TTMEA was used, with electrodes having 16 mm diameter and with spacing of 25 mm (center–center). In a first set of experiments one TTMEA was placed on the right Biceps Brachii, and signals were simultaneously recorded by two channels: medial and lateral, that is, across two couple of electrodes aligned along muscle fibers orientation (Figure 4B). Both channels had their ground, the third electrode starting from the top; the proximal and the central electrodes were therefore connected to the amplifier. EMG recordings were carried out for single‐unit isometric and phasic contractions. For the isometric contraction, the EMG signal was fed‐back to the subject by a loudspeaker and he was asked to control the muscle recruitment so that single motor unit firing was recorded; the amplifier gain was set at 10 k and filtering was in the 10 Hz–3 kHz range. For the phasic contraction, the subject was asked to produce brief explosive bursts; the amplification being at 5 k with same filtering as above. In a second set of experiments, one TTMEA was placed on the right cheek of a voluntary subject. Standard gelled electrodes (Kendall H124SG, circular shape, sensitive area diameter 16 mm) were placed on the left cheek, in almost symmetrical position. The experiment started with the facial muscles fully relaxed, then the subject produced a tonic and symmetric bilateral contraction of the Orbicularis Oris muscle group. Signals from both electrode pairs were differentially amplified (gain 5 k), filtered (10 Hz–1 kHz, with a notch at 50 Hz), and sampled (10 kHz, 12 bit). Digitized signals were full‐wave rectified and integrated, with a 200 ms running average window, to isolate 2 s of rest and another 2 s of steady tonic bilateral activation. In each unprocessed signal, the RMS amplitude was measured so as to evaluate the signal‐to‐noise ratio (activity RMS/rest RMS). Then, the power spectrum during tonic activity was calculated using the Welch's method (200 ms epochs, 50% overlap, Hanning window) and the resulting values (µV^2^ Hz^−1^) normalized to the total power in that period. The mean and median frequencies were finally calculated.

## Conflict of Interest

The authors declare no conflict of interest.

## Supporting information

Supplementary

Supplementary
