# Supplementary material for: Ultraconformable Temporary Tattoo Electrodes for Electrophysiology
Source: Adv Sci (Weinh). 2018 Jan 3;5(3):1700771. doi: 10.1002/advs.201700771 (PMC5867059; doi:10.1002/advs.201700771)
Supplement: Supplementary file 1 — Supplementary [file ADVS-5-1700771-s002.pdf]

Copyright WILEY-VCH Verlag GmbH & Co. KGaA, 69469 Weinheim, Germany, 2018.

ADVANCED SCIENCE
Open Access

Supporting Information

for *Adv. Sci.,* DOI: 10.1002/advs.201700771

Ultraconformable Temporary Tattoo Electrodes for Electrophysiology

Laura M. Ferrari, Sudha Sudha, Sergio Tarantino, Roberto Esposti, Francesco Bolzoni, Paolo Cavallari, Christian Cipriani, Virgilio Mattoli, and Francesco Greco**

Copyright WILEY-VCH Verlag GmbH & Co. KGaA, 69469 Weinheim, Germany, 2017.

Supporting Information

Title: Ultraconformable Temporary-Tattoo Electrodes for Electrophysiology

Laura Ferrari, Sudha Sudha, Sergio Tarantino, Roberto Esposti, Francesco Bolzoni, Paolo Cavallari, Christian Cipriani, Virgilio Mattoli, Francesco Greco**

Materials and Methods

Fabrication of PEDOT:PSS skin-contact electrodes - PEDOT:PSS electrodes were ink-jet printed (**Figure 1 A, B**) with a Dimatix DMP-2800 system. Customized electrodes design was implemented through a dedicated software, interfaced with the DMP software. The printing process was carried out by jetting through one or two adjacent nozzles firing at 20 kHz; drop spacing was set at 25 μm. Such parameters were optimized to minimize coffee ring effect and obtain uniform printing. After printing, electrodes were annealed in a convection oven at 110°C, for 30 minutes. The number of printed layers was varied from 1 to 10 in order to tune thickness/resistivity of printed electrodes.

Fabrication of interconnections/external contact - Depending on the selected target application and geometry of electrodes array two kinds of external connections were implemented, based on the following procedures.

Procedure 1: "Au on Glue" – planar external connectors - A scheme of fabrication is provided in **Figure S1** This assembly procedure has been used just for single electrodes. Tattoo paper glue layer (**Figure S1 A, b**) was divided into two parts, splitting the silicon paper carrier sheet from the release liner with the water based acrylic glue. The latter was laser cut in a C shape, maintaining the glue side on the top. The silicon paper was cut in an hollow C shape in order to realize a mask for the glue

layer. The mask is therefore placed above the shaped glue to allow a patterned gold deposition (**Figure S1 B, a**). At the end, a "C-shaped" Au-on-glue sheet was obtained. Au was sputtered on top of pre-cut polyimide ribbon (Kapton® film, 13 µm thick, Goodfellow), producing an Au-on-polyimide strip. The Au-on-polyimide strip was then laminated on top of the Au-on-glue sheet (**Figure S1 B, b**) fulfilling the contact from the tattoo side to the external world. The contact was finally applied on top of the printed circular electrode (**Figure S1 B, c**), with the Au side facing the PEDOT:PSS area. The glue liner, that is facing up, could be removed (**Figure S1 B, d**) and the tattoo electrode is ready to be applied on the skin.

Procedure 2: "Au on tattoo" – wired external connectors- A scheme of fabrication is provided in Figure 4 A. This fabrication and assembly procedure was usually adopted for multiple electrodes configuration (TTMEAs). The decal transfer paper (**Figure S1 A, a**) was laser cut in a rectangular shape (55x90 mm) and a design of six circular electrodes (16mm diameter, 25mm center to center distance **Figure 4 A a,b**) was printed on top of the EC layer, producing the sensible area array. An acrylic transparent sheet (55x90 mm, thickness 3 mm) was patterned in hallow C shape and linear tracks to create a physical mask. The mask was placed on top of the printed sensible area array (**Figure 4 A, c**) and gold was sputtered above it. After Au deposition the cover was lifted, freeing the patterned tattoo paper with Au interconnections from sensing area to external connections (**Figure 4 A, d**). The glue layer (**Fig. S1 A, b**) is laser cut in a rectangular shape of 55 x 90 mm with six hollow circle, the negative of the printed design. The glue sheet would provide insulation for interconnections and enable the sensing through PEDOT:PSS area, when the tattoo would be released on the skin. The silicon paper carrier sheet is removed from the glue layer and thin six copper wires (diameter 50 µm) were placed at the bottom side of the patterned acrylic glue sheet, in order to be sandwiched between the glue sheet and tattoo paper. (**Figure 4 A, e**). Free terminations of wires were usually equipped with male pins, allowing for an easy connection with the external electronics, through female pins, shielded wires and required plug.

Stretching tests. Stretching tests were performed on a conductive elastic substrate (Conductive Rubber Sheet 2-9330-02, AXEL, Japan) stretched in a controlled way through suitable linear actuators (model PI M136.CG1 by Physik Instrumente). Samples were subjected to 1% 2% and 5% strain (10 cycles for each stretch value). Stretching speed was fixed to 0.5 mm/s. Impedance measurement was performed by an LCR meter (Models E4980A by Agilent).

Electrophysiology recordings on skin with single electrodes - A pair of each kind of electrodes under investigation (tattoo electrodes and disposable Ag/AgCl by Spes Medica, model DENIS01520) were placed over the Biceps Brachii muscle of the right-handed arm. Each pair of electrodes was placed perpendicular with respect the fiber muscle direction (inter-electrode distance 20 mm; inter-pair distance 20 mm) in order to pick up signals coming from the same motor fibers (as in reference [S1]). The raw EMGs from homologous electrode pairs were collected differentially using a EMG-USB2+ amplifier (OT Bioelectronica, Torino, Italy). A reference electrode was placed on the wrist of the right forearm. Raw data were hardware filtered (high pass: 10 Hz–1st order; low pass: 900 Hz–1th order; gain: 2000), acquired on a PC (sampling frequency: 2048 kHz; resolution: 12 bits) and stored for off-line analysis. After the application of the electrodes (to let the tattoos drying), the participants were asked to cyclically (three cycles) relax and contract (isometric) the bicep, at three different contraction levels. To promote ideally constant and repetitive muscular contractions, the pressure produced by the arm on an analog pressure gauge (North Coast medical, Morgan Hill, CA) was displayed to the participant, which had to produce three fixed pressures (2, 3, and 5 pounds/inch^2, psi) (**Figure S5 A**). The recorded signals were software filtered (six order 48–52 Hz, Butterworth stop band filter) and divided in overlapping windows of 1 s (900 ms overlapped); the spectrogram of the windows was computed using a Matlab (MathWorks, Natick, MA) custom script. Furthermore, a cross-correlation analysis was performed between the recorded signals. The time domain signals picked up by the Ag/AgCl pregelled electrodes and by tattoo nanosheet electrodes matches well at all contraction lev-

els (**Figure S5 B**). A cross-correlation analysis performed on the data verified that the EMG measurement of each electrode pair was representative of the same muscle fibers activation. This analysis determined a correlation coefficient between the two signals of 0.48.

REFERENCES

[S1] J. Zuniga, T. J. Housh, C. L. Camic, C. R. Hendrix, M. Mielke, R. J. Schmidt, G. O. Johnson, *Electromyogr. Clin. Neurophysiol.* **2010**, *50*, 87.

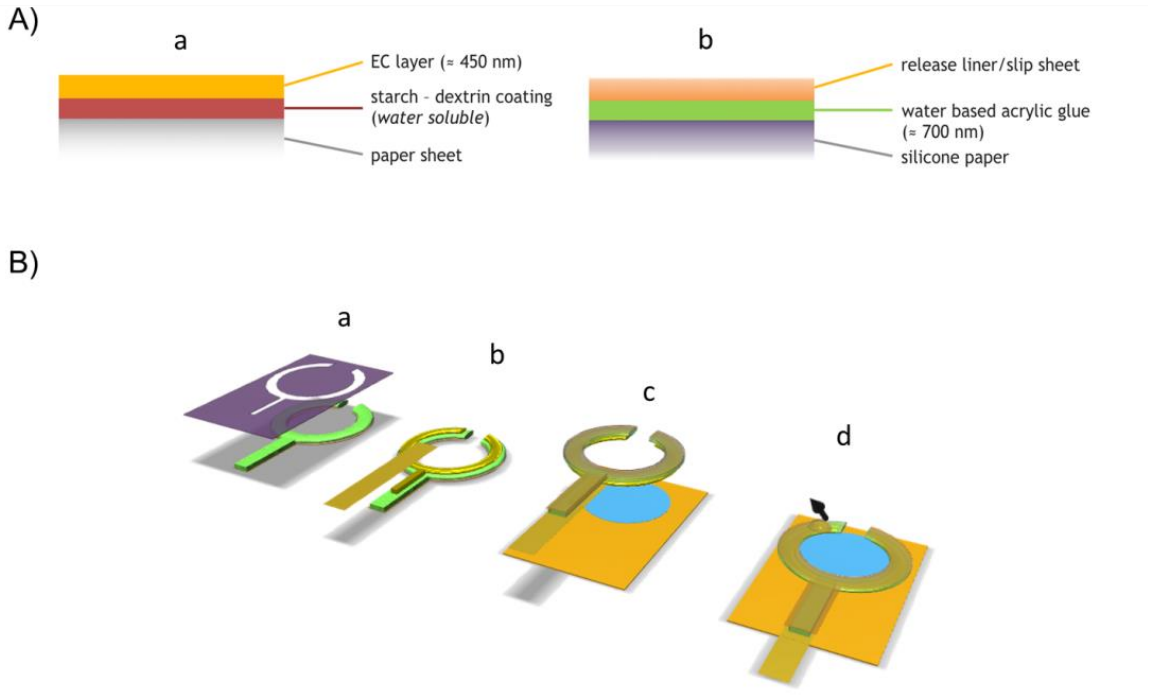

Figure S1. Schematic of multilayer electrode assembling. (A) Schematic view of a) decal transfer paper and b) glue layer used as substrates for electrodes fabrication and commercially available as "temporary transfer tattoo paper kit". **(B)** Fabrication of external contacts, «Au on glue» method for single electrodes. a) a physical mask is laser cut and placed over the glue sheet. b) Au is sputtered on top and the external contact is laminated. c) The assembly is reversed and placed on top of printed tattoo electrodes. d) The release liner is ready to be removed and the tattoo can be placed on the skin.

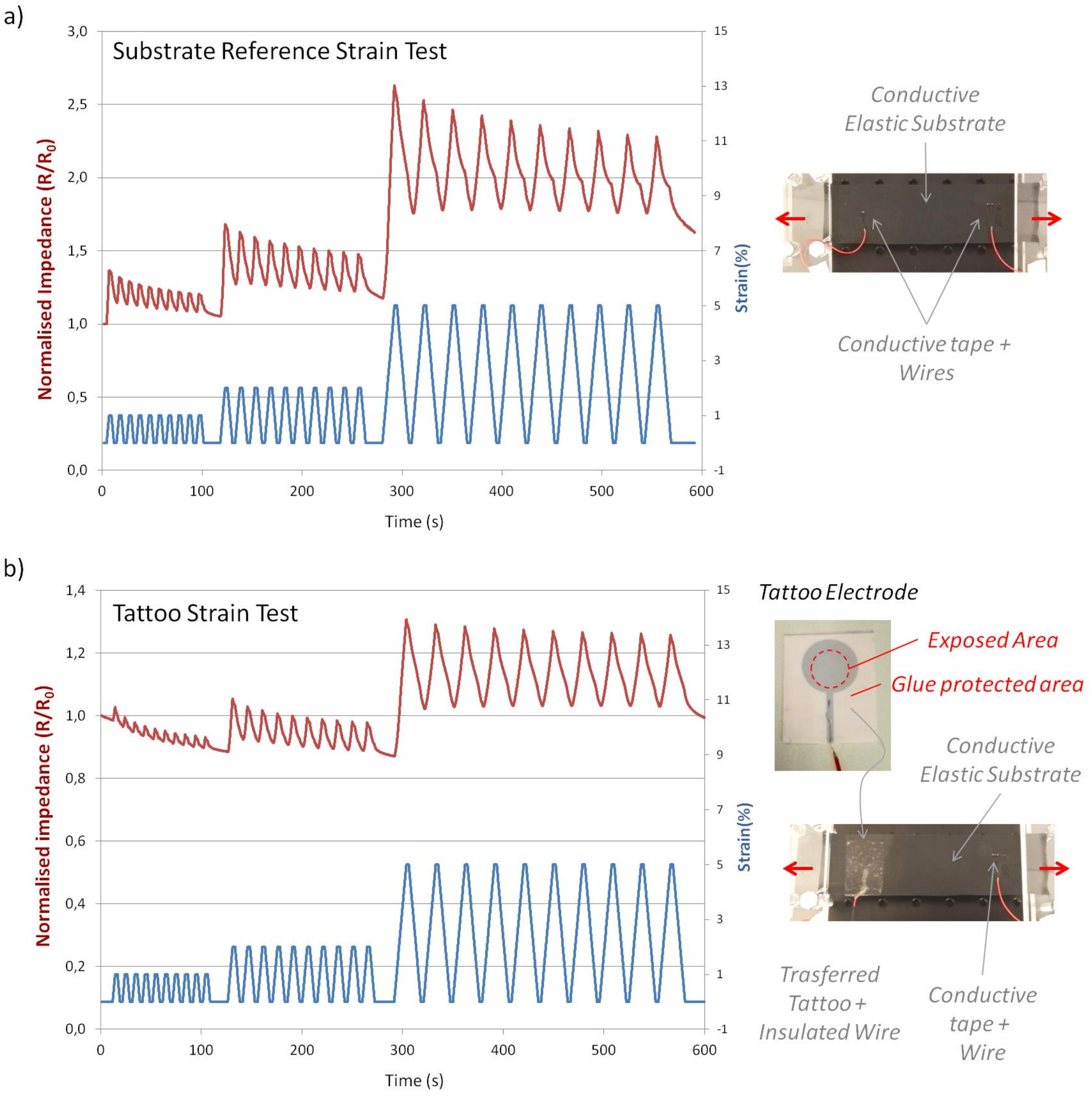

Figure S2. Repeated stretching tests. a) Reference test on the conductive elastic substrate was carried out connecting electrically two wires to the substrate by using conductive tape. b) Test on tattoo was performed connecting one wire to the substrate by conductive tape and the other one to the pre-assembled tattoo wire. Tattoo wire was insulated by tattoo acrylic glue to avoid contact with the substrate. Tests demonstrate that tattoo correctly works within 5% of stretching (reference maximum stretching for investigated skin applications).

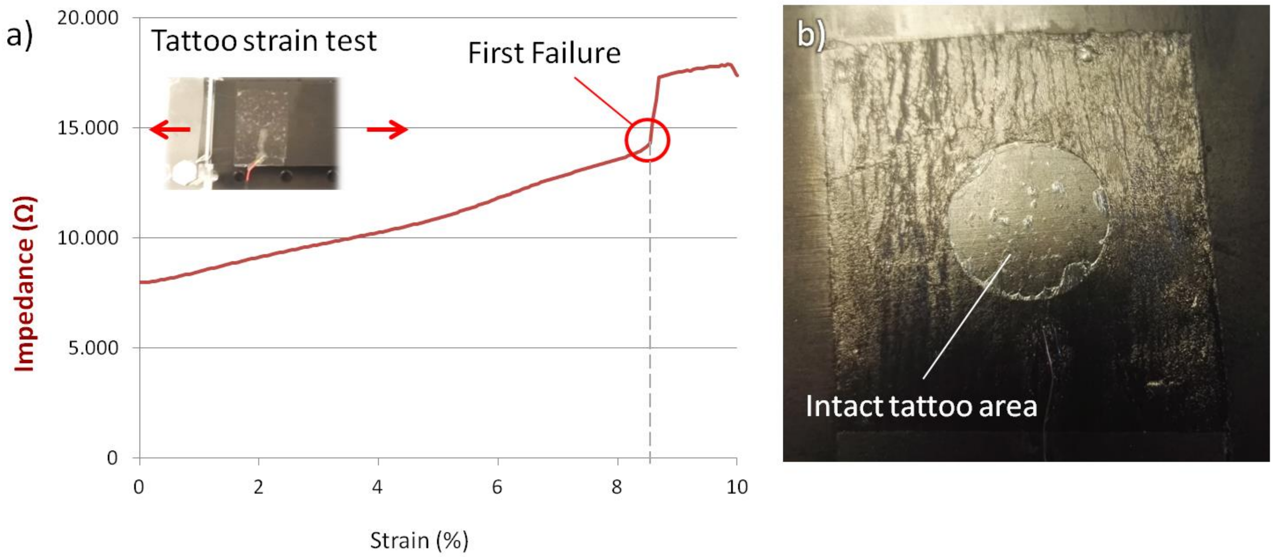

Figure S3. Stretching to failure tests. a) Tests demonstrated that tattoo can be stretched within 5% without breaking. First failures appeared over 8% of strain, as evidenced by a sudden jump in impedance reading. Nevertheless, even in this case it seems that problems could be ascribed to poor wire interconnection rather than to tattoo integrity, which is maintained even after repeated cycles at 10% of strain. b) Picture of the exposed tattoo area after several 10% strain cycles.

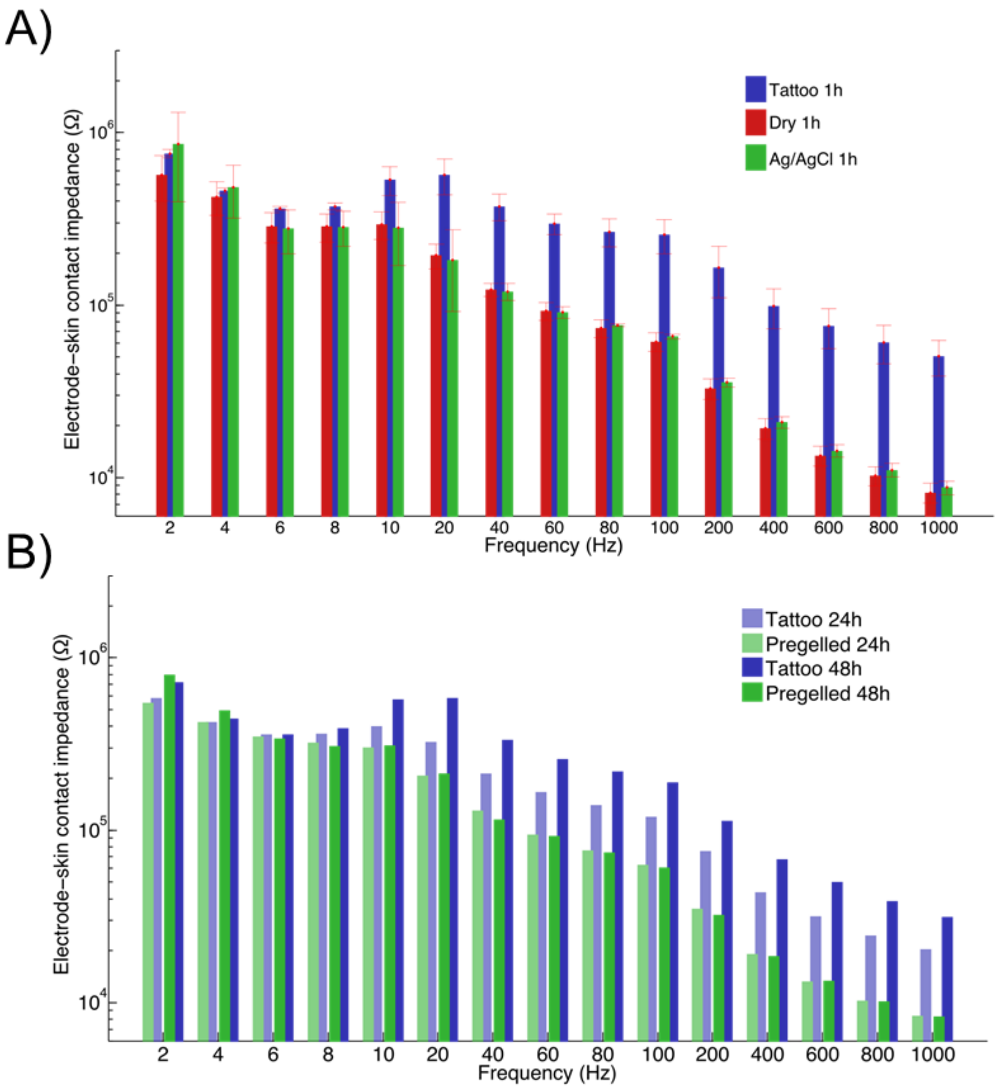

Figure S4. Impedance of the electrode-skin interface – frequency domain. (A) Impedance measured after 60 minutes from the application of the electrodes in the short-term test (7 subjects, 3 types of electrodes, 60 minutes). Bars indicate the median values (7 subjects); error bars denote the Inter Quartile Range. **(B)** Impedance recorded 24 and 48 hours after the application of the electrodes in the long-term test (1 subject, 2 types of electrodes, 48 hours).

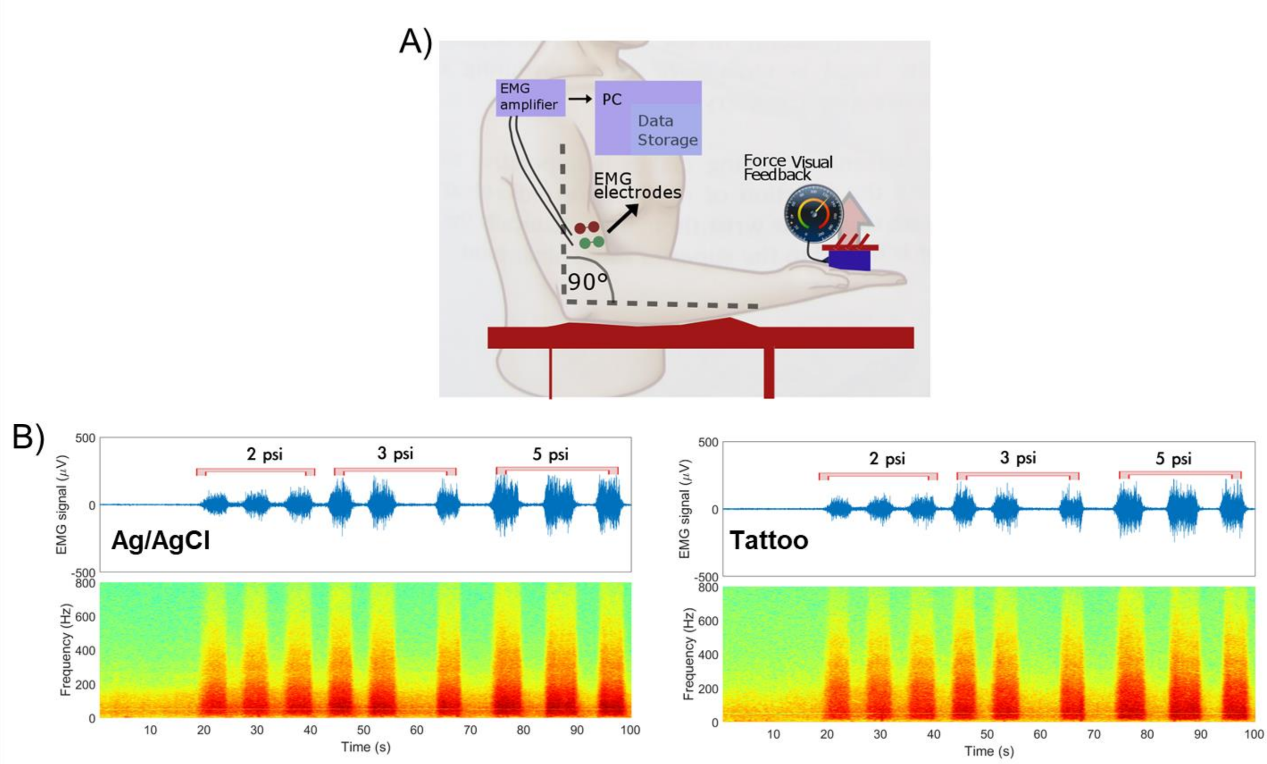

Figure S5. sEMG recordings with single tattoo electrodes. (A) Schematic of sEMG recording experiment. **(B)** Time domain (top) and spectrograms (bottom) of the EMG signals recorded during three series of contraction performed at different pressures (2, 3, 5 pounds/inch^2, psi) and rest. Signal picked up by Ag/AgCl electrodes (left), and by tattoo electrodes (right).

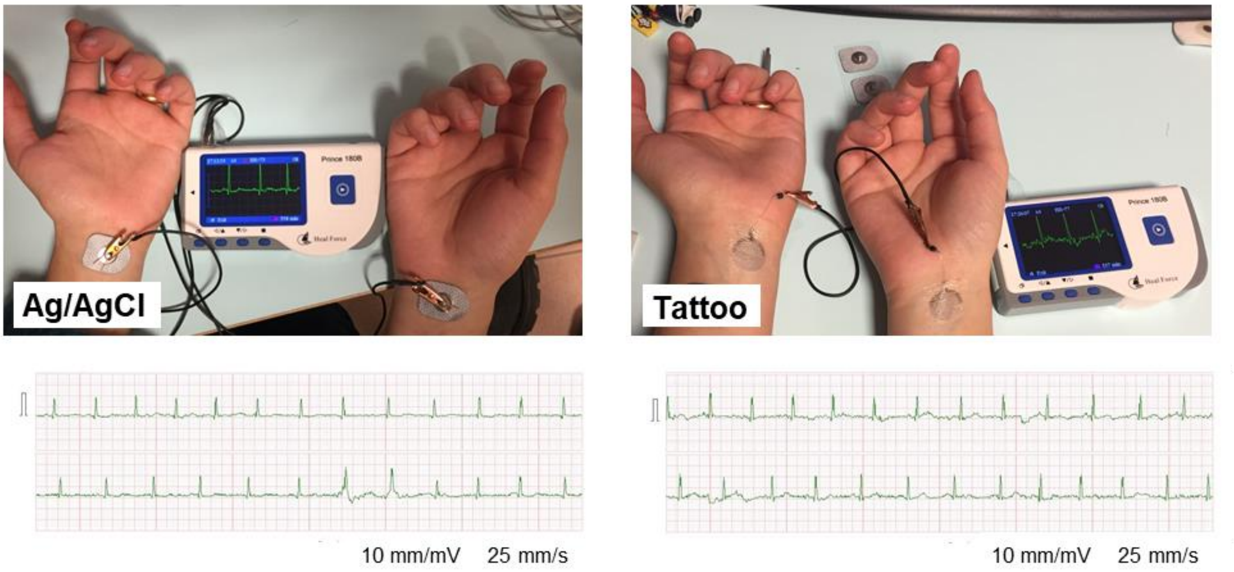

Figure S6. ECG recordings with single electrodes with a portable ECG recorder. Ag/AgCl vs. tattoo electrodes during ECG measurements with a commercial portable device.

Movie S1. Release on skin and stretchability of TTMEAs. Tattoo on a finger
